# Hybrid Mesoporous TiO_2_/ZnO Electron Transport Layer for Efficient Perovskite Solar Cell

**DOI:** 10.3390/molecules28155656

**Published:** 2023-07-26

**Authors:** Aleksandra Drygała, Zbigniew Starowicz, Katarzyna Gawlińska-Nęcek, Małgorzata Karolus, Marek Lipiński, Paweł Jarka, Wiktor Matysiak, Eva Tillová, Peter Palček, Tomasz Tański

**Affiliations:** 1 Department of Engineering Materials and Biomaterials, Silesian University of Technology, Konarskiego 18a Street, 44-100 Gliwice, Poland; tomasz.tanski@polsl.pl; 2 Institute of Metallurgy and Materials Science, Polish Academy of Sciences, Reymonta 25 Street, 30-059 Cracow, Poland; starowicz.z@imim.pl (Z.S.); gawlinska.k@imim.pl (K.G.-N.); lipinski.m@imim.pl (M.L.); 3 Institute of Materials Engineering, University of Silesia, 1a 75 Pułku Piechoty Street, 41-500 Chorzow, Poland; malgorzata.karolus@us.edu.pl; 4 Scientific and Didactic Laboratory of Nanotechnology and Material Technologies, Faculty of Mechanical Engineering, Silesian University of Technology, Towarowa 7 Street, 44-100 Gliwice, Poland; wiktor.matysiak@polsl.pl; 5 Department of Materials Engineering, Faculty of Mechanical Engineering, University of Žilina, Univerzitná 1 Street, 010 26 Zilina, Slovakia; eva.tillova@fstroj.uniza.sk (E.T.); peter.palcek@fstroj.uniza.sk (P.P.)

**Keywords:** photovoltaics, perovskite solar cell, electron transport layer, nanostructures

## Abstract

In recent years, perovskite solar cells (PSCs) have gained major attention as potentially useful photovoltaic technology due to their ever-increasing power-conversion efficiency (*PCE*). The efficiency of PSCs depends strongly on the type of materials selected as the electron transport layer (ETL). TiO_2_ is the most widely used electron transport material for the n-i-p structure of PSCs. Nevertheless, ZnO is a promising candidate owing to its high transparency, suitable energy band structure, and high electron mobility. In this investigation, hybrid mesoporous TiO_2_/ZnO ETL was fabricated for a perovskite solar cell composed of FTO-coated glass/compact TiO_2_/mesoporous ETL/FAPbI_3_/2D perovskite/Spiro-OMeTAD/Au. The influence of ZnO nanostructures with different percentage weight contents on the photovoltaic performance was investigated. It was found that the addition of ZnO had no significant effect on the surface topography, structure, and optical properties of the hybrid mesoporous electron-transport layer but strongly affected the electrical properties of PSCs. The best efficiency rate of 18.24% has been obtained for PSCs with 2 wt.% ZnO.

## 1. Introduction

Rapid advances in materials technology are creating many novel solutions for energy-efficient applications in solar energy [1,2,3,4,5]. The selection of materials is the foundation of all engineering applications and design. This selection process can be defined by application requirements, possible materials, physical principles, and selection [6,7,8,9]. The intended results of the material selection process lead to the identification of one or more materials with properties that meet the functional requirements of a product. The material selection process is one of the basics of design and engineering [10,11]. The second half of the 20th century brought significant progress in the field of material science, nanotechnology, and materials processing. These developments have led to the production of materials targeted at providing solutions in various key areas, including photovoltaics. Progress in this area is additionally due to the increasing awareness of potential ecological collapse, energy insecurity, and the rising cost of living. Emerging solar-cell technologies that use advanced materials, such as perovskite, dye, metal oxide, organic materials, and quantum dots, are the answer to the challenges of conversion efficiency and longevity [1,12,13].

Since the initial reports on perovskite solar cells (PSCs) with an efficiency of 3.8% in 2009 [14], there has been a rapid increase in reported efficiencies [15,16,17,18,19,20]. The certified stabilized power-conversion efficiency of single-junction perovskite solar cells (PSCs) has reached a value of 26% which is comparable with that of monocrystalline silicon solar cells [21].

Depending on the light incidence, PSCs can be generally divided into either n-i-p (regular) or p-i-n (inverted) geometry [22,23], where [24,25]:

- n refers to n-type—electron transport layer (ETL),
- i refers to the perovskite optical absorption layer,
- p refers to p-type—hole transport layer (HTL).

The electron transport layer (ETL) in PSCs plays an indispensable role in collecting and transporting photogenerated electron carriers and serves as a hole-blocking layer, thus realizing effective charge separation and suppressing charge-carrier recombination. The ETL in PSCs should possess [25,26,27,28,29,30,31]:

- a proper energy-level alignment with the perovskite layer;
  ETL should have the lowest unoccupied molecular orbital (LUMO) and highest occupied molecular orbital (HOMO), lower than the perovskite active layer. The cascading energy structure ETL can improve electron transport to the cathode, suppress back recombination, and enhance device effectiveness.
- a wide bandgap to ensure good transmittance in the visible light range;
- high electron mobility (>2.5 × 10^−5^ cm^2^ V^−1^ s^−1^) for efficient electron transport within the ETL;
- a good photochemical stability.

Moreover, the ETL in n-i-p geometry serves as a nucleation site for the perovskite, which affects the crystal growth and hence the PSC efficiency [32,33].

Many n-type semiconductors, including both organic and inorganic materials, have been employed as ETLs. Organic ETMs (e.g., fullerene or its derivatives, small molecule 3,9-bis(2-methylene-(3-(1,1-dicyanomethylene)-indanone)-5,5,11,11-tetrakis(4-hexylphenyl)-dithieno[2,3-d:2′,3′-d′]-s-indaceno[1,2-b:5,6-b′]-dithiophene (ITIC)) are usually employed in inverted p-i-n PSCs [30,34,35]. In conventional n-i-p structure devices, inorganic ETMs such as TiO_2_ [36], ZnO [37], SnO_2_ [38,39], Nb_2_O_5_ [40], WO_3_ [41], BaSnO_3_ [42], and Zn_2_SnO_4_ [33] are more commonly used. Each material has specific benefits to increase the solar cell efficiency. In general, organic materials have lower production costs, but inorganic materials generally have higher thermal and long-term stability [32].

Nowadays, typical PSCs are generally fabricated with a mesoscopic or planar architecture. In a planar architecture, each layer is deposited as a dense, thin film. While in a mesoscopic architecture, perovskite is adsorbed on a mesoporous scaffold. The perovskite grain growth is limited by the pore size of the mesoporous layer, but the thicker perovskite layer provides better light harvesting. If the scaffold layer is involved in the electron transfer, it is referred to as active (e.g., TiO_2_, ZnO, and SnO_2_) and named mesoporous electron transport layer; otherwise, it is passive (e.g., SiO_2_, Al_2_O_3_, ZrO_2_) [43,44,45,46].

TiO_2_ is the most widely used electron-transport material for the n-i-p structure of PSCs. Titanium dioxide nanostructures play a crucial role in the extraction of photoinduced electrons from the perovskite and then in their transport to the electrode, both as a compact and mesoporous layer. This semiconductive material has a good band alignment with perovskite, which enables faster electron injection from the active layer. Nevertheless, TiO_2_ suffers from low electron mobility and high defect-state density, which limits the overall device performance [47,48].

A potential n-type semiconducting material is zinc oxide (ZnO) from group II–VI with a band gap energy of 3.37 eV and an exciton binding energy of 60 meV at room temperature [49,50]. Moreover, ZnO has unique chemical and physical properties, such as a good thermal and chemical stability, a high electrochemical coupling coefficient, piezoelectricity, and a broad scope of radiation absorption and high photostability, which provide a wide range of applications in various fields and make it one of the crucial technological materials among all metal oxides. ZnO crystallizes in two main forms, cubic zinc-blende and hexagonal wurtzite (B4). The latter is the most thermodynamically stable crystal structure and the most common in ambient conditions. The zinc-blende form can be stabilized by growing ZnO on substrates with a cubic lattice structure [49,51,52]. 

Recent studies have shown a variety of ZnO nanostructures, such as nanotetrapods, nanomultipods, nanobelts, nanotubes, nanoparcicles, nano-flowers, nanowires, nanorods, nanoribbons, nanorings, nanoneedles, nanosheets, and shuttle- and comb-like. Over the last few years, scientists have focused on the fabrication and application of one-dimensional (1D) nanostructure materials, such as nanowires and nanorods, because of their fundamental importance and the wide range of potential applications, e.g., in nanodevices [53,54,55].

It is essential to fabricate an electron-transport layer with a suitable composition to improve charge-carrier extraction and transportation for achieving a higher efficiency of solar cells. In this study, we introduce the ZnO nanopowder, consisting of various shape nanostructures, to a TiO_2_ solution for the fabrication of the mesoporous electron transport layer of PSCs. The regular (n-i-p) mesoscopic architectures of perovskite solar cells were studied. In order to produce the mesoporous hybrid TiO_2_/ZnO-layer precursor was prepared by dissolving TiO_2_ paste in ethanol. Then, contents of different weights of ZnO nanostructures (0, 1, 2, 3, 4 and 8 wt.%) were added to the TiO_2_ solution. The prepared mesoporous ETLs were characterized by scanning electron microscopy, atomic force microscopy, X-ray diffraction, and UV-Vis spectroscopy. The effects of using ZnO nanostructures with various shapes and dimensions on the electrical properties of PSCs were also investigated.

## 2. Technology of Perovskite Solar Cells

In the present study, we investigated the perovskite solar cell in a structure of FTO/compact TiO_2_ (c TiO_2_)/mesoporous TiO_2_ (mp-TiO_2_) with the addition of ZnO nanostructures/FAPbI_3_/2D perovskite/Spiro-OMeTAD/Au. The perovskite devices were prepared according to the procedure developed in the Institute of Metallurgy and Materials Science of the Polish Academy of Sciences. Fluorine-doped tin-oxide-coated glass was ultrasonically cleaned sequentially in an aqueous solution of 2% Hellmanex, deionized water, isopropanol for 5 min in each solvent, and then dried. The TiO_2_ dense layer have been deposited from tetraethyl orthotitanate, dissolved in a mixture of ethanol and hydrochloric acid, using spin-coating method. Then, the TiO_2_ thin film was dried at 200 °C and heated at 500 °C. In order to produce the mesoporous layer, a TiO_2_ precursor solution was prepared by dissolving 30 NR-D paste in ethanol. Different weight contents of ZnO nanostructures (x = 0, 1, 2, 3, 4 and 8 wt.% to weight of TiO_2_) were added to the TiO_2_ solution. The spin-coating method was used to create the mesoporous layer of TiO_2_/ZnO. The samples were dried at 200 °C and heated at 500 °C.

The FAPbI_3_ perovskite precursor was produced in a glovebox filled with nitrogen. The precursor solution was developed by mixing lead iodide (PbI_2_), formamidinium iodide (FAI) and methylammonium chloride (MACl) in a co-solvent of DMF/DMSO (4:1 *v*/*v*). The FAPbI_3_ perovskite layer was deposited using the anti-solvent method with ethyl acetate. The prepared perovskite precursor solution was deposited on the meso-TiO_2_/ZnO-coated substrate at 8000 rpm (with an acceleration of 2000 rpm). Diethyl ether was dropped onto the substrate during the 10th s of the spin-coating. The perovskite film layer was annealed at 150 °C for 10 min. to allow the formation of a black phase FAPbI_3_. To fabricate a 2D perovskite, 0.04 M octylammonium iodide (OAI) solution was prepared by dissolving OAI in IPA. OAI solution was deposited on the perovskite substrate by spin-coating at 3000 rpm for 15 s, and then the substrate was heated at 100 °C.

Spiro-OMeTAD was used as the hole transport layer (HTL) material. Spiro-OMeTAD was dissolved in chlorobenzene and mixed with LiTFSI solution (prepared by dissolving LiTFSI in acetonitrile) and 4-tert-butylpyridine (tBP). Spiro-OMeTAD solution was spin-coated on the perovskite layer at 2000 rpm for 30 s. After that, the samples were taken out of the glove box, masked, and coated with gold by thermal evaporation. Au electrodes had a surface of 0.25 cm^2^ and a thickness of approximately 80 nm.

## 3. Results and Discussion

**Figure 1:**
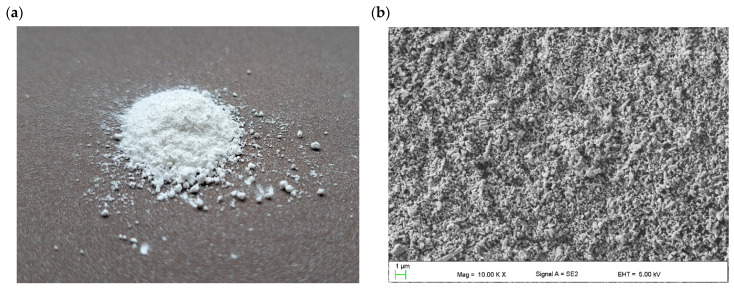

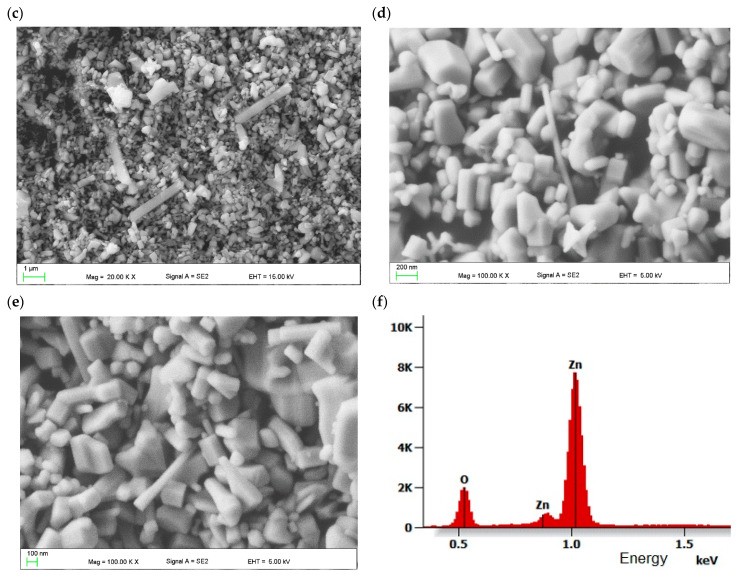
Morphology of ZnO nanopowder: (**a**) macroscopic image (**b**–**e**) SEM images and (**f**) EDS analysis of the chemical composition.

Figure 1 presents the morphology of the ZnO nanopowder used to fabricate the hybrid TiO_2_/ZnO mesoporous electron-transport layer. The ZnO nanopowder consists of nanostructures of various shapes, including nanoparticles and nanorods/nanowires (Figure 1b–e). The EDS analysis of the chemical composition certified the purity of the ZnO nanopowder and showed the presence of the two elements zinc and oxygen. The applied ZnO material is described in detail in the manuscripts [50,54,55,56,57]. The XRD analysis carried out for the nanopowder showed the existance of sharp crystalline peaks for 2θ angles: 37.1°, 40.2°, 42.4°, 55.8°, 66.8°, 74.6°,80.9° and 82.4° originating from Miller indices: (010), (002), (011), (012), (110), (013), (112) and (021), respectively. These peaks indicate the existance of hexagonal ZnO phase characteristzed by the P63mc space group (ICDD PDF4+ 98-018-5827) [54]. In addition, electron diffraction on a selected area (SAED) was carried out using a transmission electron microscope, which confirmed the results of the study of the structure of the ZnO nanopowder obtained using XRD and showed that the examined nanostructures were single crystals [54]. The analysis of the morphology of the tested ZnO semiconductor nanopowder, based on the recorded TEM and SEM images, showed the spherical and oblong shape of the tested nanostructures, where their diameters ranged from about 50 nm to 350 nm, and lengths reached about 500 nm [50,54,55]. Studies of the optical properties of the ZnO nanostructures employed were made using UV-Vis spectroscopy. Analysis of the absorption spectrum as a function of the wavelength showed a sharp absorption edge-fall at 360 nm wavelength, while the absorption maximum fell at 340 nm wavelength, which is confirmed with the results obtained for pure one-dimensional ZnO nanostructures shown in [56]. The energy gap (*E_g_*) analysis based on the obtained UV-Vis spectrum showed that the investigated ZnO nanostructures were characterized by an *E_g_* value of about 3.2 eV [57].

**Figure 2:**
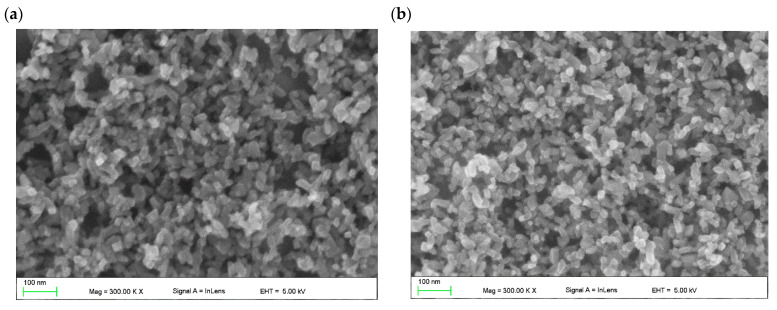
Topography of spin-coated mesoporous TiO_2_ layer (**a**) without and (**b**) with 8 wt.% addition of ZnO nanostructures.

Figure 2 shows SEM topography of the spin-coated TiO_2_ layer with and without the addition of ZnO nanostructures. A highly porous structure without cracks and gaps can be observed. The metal oxide nanostructures form agglomerates. It was found that the addition of ZnO up to 8 wt.% does not influence the mesoporous ETL surface topography.

**Figure 3:**
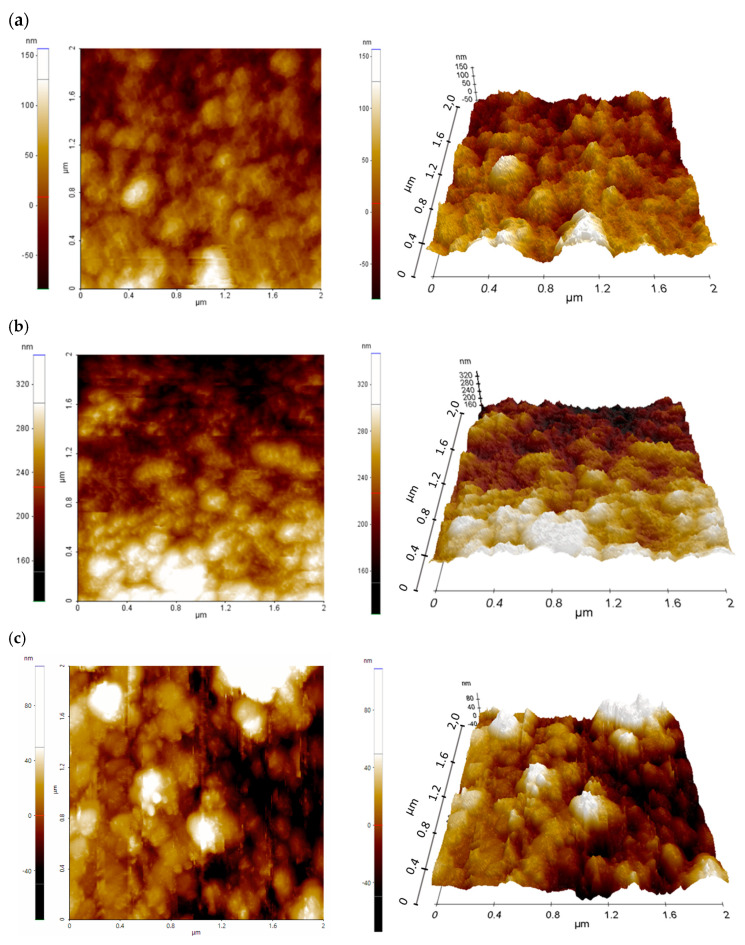

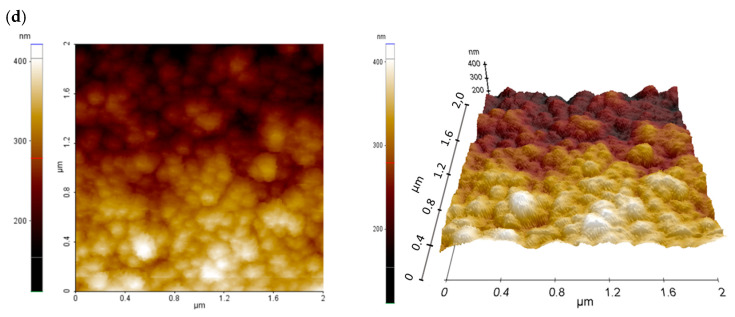
2D and 3D AFM images of mesoporous TiO_2_ layer (**a**) without and (**b**) with 2% (**c**) with 4% (**d**) with 8% addition of ZnO nanostructures.

The surface topography of a deposited hybrid TiO_2_/ZnO layer was also studied using an atomic-force microscope in non-contact mode (Figure 3). A quantitative representation of the surface topography of the pure mesoporous TiO_2_ layer with the addition of ZnO nanostructures is represented by the roughness coefficients: root mean square (*RMS*) and the average arithmetic deviation of the profile from the average line (*Ra*) (Table 1).

The mesoporous TiO_2_ layer is reflected in the relatively high *RMS* and *Ra* values. The AFM images clearly indicate a structure composed of nanoparticles and its agglomerates with a size above 100 nm, forming three-dimensional complex structures with a high specific surface area. The large specific surface area, in turn, plays an important role in the penetration of the perovskite solar-radiation absorber, and thus improves the efficiency of the final solar cell.

It can be seen that the share of nanostructural ZnO nanoadditives does not significantly affect the surface area of the layers obtained in the case of 1–4% share. It can be noticed that the layer with the highest content of ZnO nanoaddition shows significantly higher values of *Ra* and *RMS* coefficients, which may improve the charge transport in the perovskite device.

**Figure 4:**
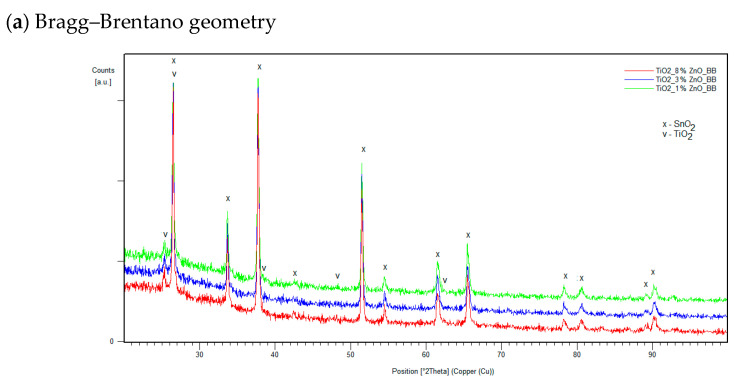

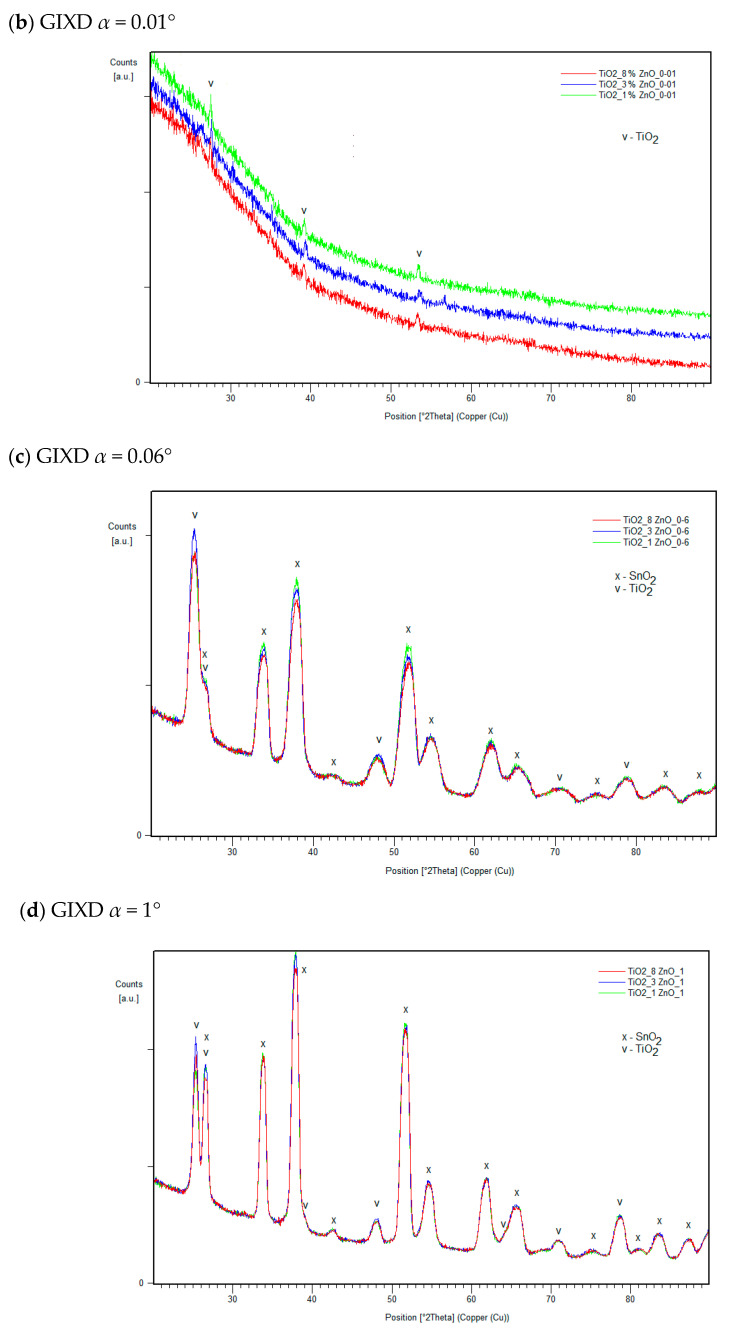
Examples of diffraction patterns of manufactured layers with the weight content of 1%, 3% and 8% ZnO for measurements in the Bragg–Brentano geometry (**a**) and at the GIXD angles *α* = 0.01° (**b**), *α* = 0.6° (**c**) and *α* = 1° (**d**).

It was found that the hybrid mesoporous TiO_2_/ZnO electron-transport layer with extreme ZnO content (1% and 8% ZnO) and one with intermediate content (3% ZnO) selected for testing are characterized by identical diffraction patterns. This proves the lack of structural differences in the tested layers. Example diffraction patterns obtained for three samples with different compositions at different GIXD angles are shown in Figure 4. The top layer (*α* = 0.1°) does not differ from the layers tested at the angle *α* = 0.1°, which indicates the lack of additional oxide layers that could be formed due to the atmosphere on the material’s surface.

**Figure 5:**
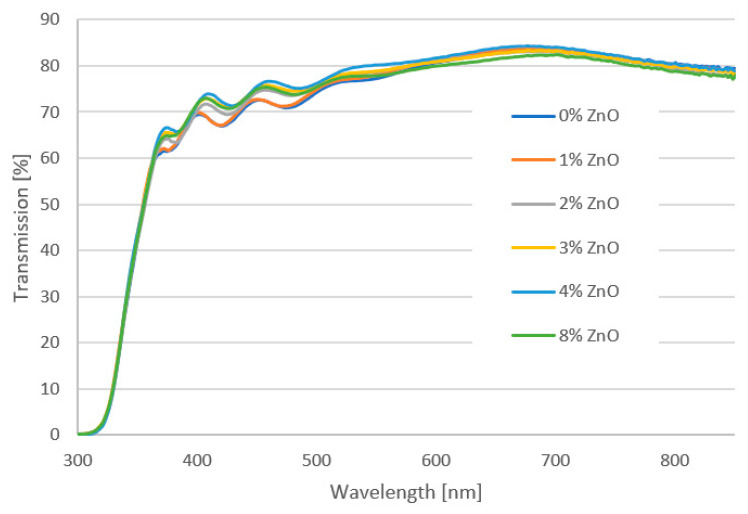
UV-Vis transmission spectra of the hybrid mesoporous TiO_2_/ZnO layer with different addition of ZnO nanostructures.

**Figure 6:**
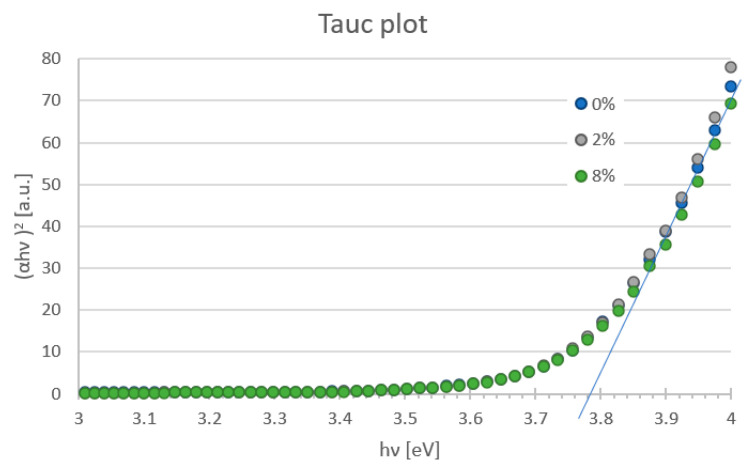
Band gap energy of the mesoporous TiO_2_ layer with different addition of ZnO nanostructures estimated from Tauc plot.

Figure 5 presents the transmittance plot of tested mesoporous titanium oxide layers with the addition of zinc oxide nanostructures in 0%, 1%, 2%, 3%, 4% and 8% deposited on FTO glass with 70 nm-thick blocking TiO_2_. It was found that the incorporation of ZnO nanostructures into the mesoporous TiO_2_ layer does not significantly affect light transmission. All produced layers show a transmittance above 60% for wavelengths in the range of 366–900 nm. It was observed that for higher contents of ZnO (2%, 4% and 8%), for the wavelength below 500 nm, there is a peak shift. This may indicate an increase in the thickness of the spin-coated layers. The Tauc plot method was used to determine the band-gap energy of manufactured films. The absorption coefficient (*α*) was calculated from the formula: *α* = −(ln(*T*/(1 − *R*))/*d* where: *d*—thickness of the tested layer, *T*—transmission at a given wavelength, *R*—reflection coefficient. In the Tauc plot, the direct optical band gap can be determined from the intercept of the leading-edge linear extrapolation with hν axis, as displayed by lines in Figure 6. The calculated *E_g_* is around 3.78 eV for all tested samples. This response is dominated by FTO or glass substrate, but no important differences were visible for any ZnO addition layers. The analysis of transmittance spectra and extracted optical band gaps suggests that the addition of ZnO nanostructures to the mesoporous TiO_2_ does not worsen the optical properties but might even be slightly beneficial. It confirms that a hybrid TiO_2_/ZnO layer is a promising candidate as the mesoporous ETL, owing to its high transparency and appropriate band gap that is well fitted into the energy structure of the perovskite solar cell.

**Figure 7:**
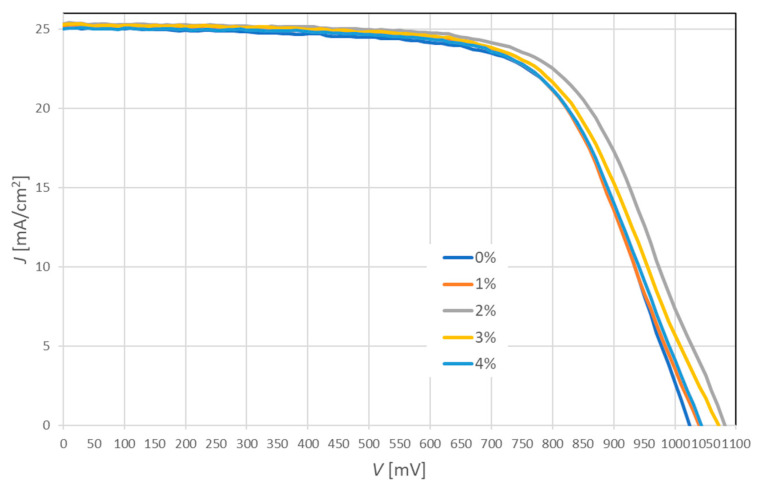
Average current-voltage characteristics of perovskite solar cells based on hybrid mesoporous TiO_2_/ZnO layer with a different addition of ZnO nanostructures.

The measurement of the current-voltage *(J-V)* characteristics is the most important step for quality control and optimization of the fabrication process in research and industrial production of solar cells. A comparison of the *J-V* characteristics of the PSCs with a hybrid mesoporous TiO_2_/ZnO layer is shown in Figure 7. The electrical properties such as power conversion efficiency (*PCE*), fill factor (*FF*), short-circuit current density (*J_sc_*) and open-circuit voltage (*V_oc_*) of fabricated PSCs are summarized in Table 2. In this study, four series of perovskite solar cells were fabricated. After the first series, PSCs with an ETL layer containing 8% ZnO were abandoned due to a significant decrease in efficiency.

It was found that the incorporation of ZnO nanostructures into the mesoporous TiO_2_ layer has a major impact on the efficiency of PSCs. The highest efficiency of 18.24% demonstrated solar cells with the addition of 2% ZnO, which increased by 1.13% compared to devices with pure TiO_2_ mesoporous layer. The obtained research results indicate that the efficiency of PSCs is mainly determined by the open-circuit voltage. The addition of ZnO can result in better band alignment with perovskite, which makes it possible for faster electron injection from the active layer. The highest fill factor of 0.67 was obtained for PSCs with pure TiO_2_ and with the addition of 2% ZnO nanostructures. The short-circuit current density rises slightly (by 0.3 mA/cm^2^) with an increasing amount of ZnO addition to 2% from 25.09 (without ZnO) to 25.39 mA/cm^2^ (2% ZnO). This may indicate that the formation of local TiO_2_-ZnO heterostructures promotes faster electron transport and increases the number of carriers. Despite the significant improvement in efficiency by the addition of ZnO, it was noted that its excessive concentration deteriorated the photovoltaic performance of PSCs. This may be because higher ZnO concentration increases the resistance to the electron transport and charge transition, which promotes the charge-recombination process.

**Figure 8:**
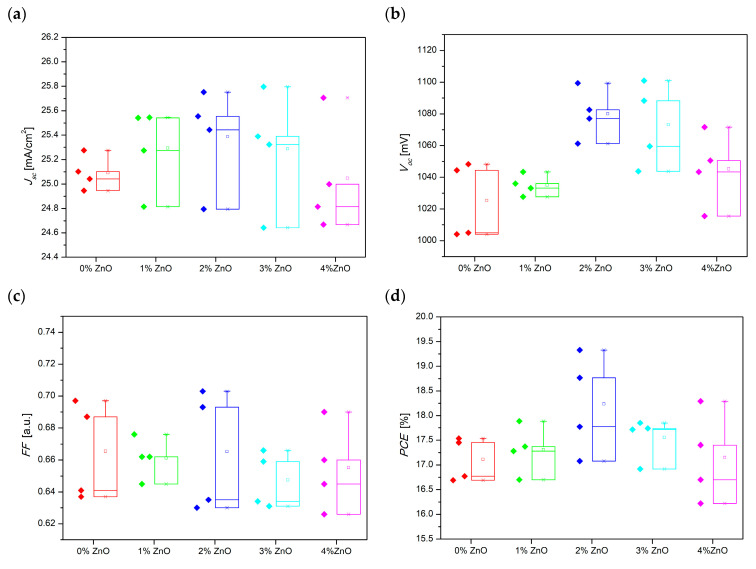
Box and whisker plots for electrical properties of prepared PSCs based on a hybrid mesoporous TiO_2_/ZnO layer with different addition of ZnO nanostructures (**a**) *J_sc_*, (**b**) *V_oc_*, (**c**) *FF*, (**d**) *PCE*.

As we have mentioned earlier, four series of perovskite solar cells were prepared. Figure 8 presents the repeatability and reproducibility analysis of manufactured perovskite solar cells by means of box and whisker plots. It was observed that the highest repeatability of *FF* and efficiency were found for the PSC with 1% and 3% ZnO, respectively. Solar cells with the addition of 3 and 4% ZnO seem to show a significant dispersion in the obtained short-circuit current density compared to other devices. However, the standard deviation for them does not exceed 1.99%. Moreover, the close distribution of the *J_sc_* and *V_oc_* (<2.42%) may also indirectly indicate the homogeneity of the deposited layer. The values of the electrical properties of the devices showed only a slight deviation from the average, which proves the high repeatability of the produced PSCs.

## 4. Materials and Methods

Ultra-pure lead iodide beads were purchased from Alfa Aesar. Formamidinium iodide and methylammonium chloride were purchased from Greatcell Solar Ltd. (Queanbeyan, Australia). Spiro OMeTAD(N^2^,N^2^,N^2^′,N^2^′,N^7^,N^7^,N^7′^,N^7′^-octakis(4-methoxyphenyl)-9,9′-spirobi[9H-fluorene]-2,2′,7,7′-tetramine), ultra-dry dimethylformamide (DMF), ultra-dry dimethyl sulfoxide (DMSO) and ultra-dry chlorobenzene (CB), dry isopropanol (IPA), 4-tert-butyl pyridine, and lithium bistrifluorosulfonylimide (LiTFSI) were purchased from Sigma Aldrich. All the chemicals were used as received without additional purification. Fluorine-doped tin oxide (FTO)-coated glass (8 Ω/sq) and titanium dioxide paste (30 NR-D) were purchased from Greatcell Solar Ltd. (Queanbeyan, Australia). The applied ZnO nanopowder is described in detail in the manuscripts [50,54,55,56,57], but in this paper the morphology of the ZnO nanopowders and their chemical composition and the topography of the hybrid TiO_2_/ZnO layer were examined using the Supra 35 scanning electron microscope (SEM) from Carl Zeiss (Jena, Germany), equipped with an EDS detector. The surface topography of the spin-coated electron transport layer was investigated using an atomic force microscope (AFM) (Park Systems XE 100 with dedicated XEI Software 5.2.4 Build 1 (Suwon, Republic of Korea) in non-contact mode.

X-ray measurements of selected samples containing 1%, 3% and 8% (weight) of ZnO were performed on a PANalytical Empyrean Diffractometer (Malvern, UK), using copper radiation (Cu K*α* = 1.5418 Å) and a PIXcell counter. The diffraction patterns of each sample were measured in the Bragg–Brentano geometry (as a reference, substrate measurement) and in the Grazing Incident X-ray Diffraction geometry for different angles of incidence of the X-ray beam for the analysis of individual layers. The effective penetration depth was estimated on the basis of the following formula:

(1) $$z=\frac{-ln(1-0.05)}{\mu \;[\frac{1}{\sin\alpha }+\frac{1}{\sin\left(2\theta -\alpha \right)}]}$$

where:

*z*—effective penetration depth,

*µ*—sum of linear X-ray absorption coefficients of chemical compounds present in the tested material,

*α*—angle of incidence of the X-ray beam,

2*θ*—angle at which the diffraction peak was recorded, taking into account the copper radiation, the appropriate angle of incidence of the radiation, the absorption coefficients of the components of the layers (Table 3). Estimated radiation-penetration depths may differ from the actual ones due to the porosity of the material, heterogeneity of layers, and local differences in material density. For this reason, measurements were made for a number of angles, not limited to the expected layer thicknesses.

A Lambda 950 S UV-Vis spectrophotometer from Perkin Elmer (Waltham, MA, USA) was used to determine the optical properties of the manufactured hybrid TiO_2_/ZnO electron-transport layers in the wavelength range of 300–900 nm. The electrical parameters of manufactured perovskite solar cells with a hybrid mesoporous TiO_2_/ZnO electron transport layer were characterized by measurements of current-voltage (*I*–*V*) characteristics using PV Test Solutions Tadeusz Zdanowicz Solar Cell I-V Tracer System (Wroclaw, Poland), Keithley 2400 source meter (Cleveland, OH, USA), Photo Emission Tech (Ventura, CA, USA) AAA class solar simulator under standard AM 1.5 radiation and light intensity 1000 W/m^2^.

## 5. Conclusions

Due to growing energy consumption, uncertainty about its supply, and the limited resources of fossil fuel, it is becoming necessary to convert energy from renewable sources. Solar power is an important resource because of its inexhaustibility and pollution-free character. Moreover, solar energy is the cleanest and most abundant renewable energy source available in the world. The answer to these challenges lies in solar cells that directly convert solar energy into electricity. Perovskite solar cells have attracted tremendous attention thanks to their good stability and rising power-conversion efficiency.

In this paper, TiO_2_ nanoparticles with the addition of various ZnO nanostructure, including nanoparticles and one-dimensional nanoadditives such as nanorods and nanowires, were proposed as a hybrid mesoporous electron transport layer of PSCs. The introduction of ZnO nanostructures with various shapes and dimensions into the mesoporous TiO_2_ layer does not negatively affect its transparency. X-ray diffraction analysis does not reveal a change in the structure of layers with the addition of ZnO nanopowder (1, 3, 8 wt.%). The highest efficiency of 18.24% was obtained for perovskite solar cells with the addition of 2 wt.% ZnO, which is 1.13% higher compared to devices without ZnO nanostructures. This is probably due to the higher electron mobility compared to the TiO_2_ material and the smooth path for electron transport in one-dimensional structures. The addition of ZnO can result in better band alignment with perovskite, which makes it possible for faster electron injection from the active layer. The statistical analysis showed good reproducibility and repeatability of the photovoltaic parameters of the manufactured devices. These results indicate that the ZnO/TiO_2_ composite has demonstrated to be a promising candidate as a mesoporous electron transport layer for high-performance mesoscopic perovskite solar cells.

## Figures and Tables

**Table 1:** Comparison of roughness values of mesoporous TiO_2_ layer without and with addition of ZnO nanostructures.

Roughness Values	0% ZnO	1% ZnO	2% ZnO	3% ZnO	4% ZnO	8% ZnO
*RMS* [nm]	34	28	39	26	25	57
*Ra* [nm]	21	23	32	21	19	49

**Table 2:** Average electrical parameters and their standard deviation for perovskite solar cells based on hybrid mesoporous TiO_2_/ZnO layer with different addition of ZnO nanostructures.

wt.% of ZnO	*J_sc_* [mA/cm^2^]	*V_oc_* [V]	*FF*	*PCE* [%]
0	25.1 ± 0.1	1025 ± 24	0.67 ± 0.03	17.11 ± 0.44
1	25.3 ± 0.3	1035 ± 7	0.66 ± 0.01	17.31 ± 0.49
2	25.4 ± 0.4	1080 ± 16	0.67 ±0.04	18.24 ± 1.00
3	25.3 ± 0.5	1073 ± 26	0.65 ± 0.02	17.56 ± 0.43
4	25.1 ± 0.5	1045 ± 23	0.66 ± 0.03	17.15 ± 0.90

**Table 3:** Measurement condition in GIXD.

Incidence angle in GIXD geometry *α* [°]	0.01	0.1	0.2	0.3	0.4	0.5	0.6	0.7	0.8	0.9	1	2	3	BB (Bragg Brentano)
Penetration depth *z* [μm]	0.006	0.06	0.12	0.18	0.24	0.3	0.36	0.42	0.48	0.54	0.6	1.2	1.8	35

## Data Availability

Not applicable.
